# Can a Trained Radiology Technician Do Arterial Obstruction Quantification in Patients With Acute Pulmonary Embolism?

**DOI:** 10.3389/fcvm.2019.00038

**Published:** 2019-04-10

**Authors:** David C. Rotzinger, Stéphane Breault, Jean-François Knebel, Catherine Beigelman-Aubry, Anne-Marie Jouannic, Salah D. Qanadli

**Affiliations:** ^1^Cardiothoracic and Vascular Division, Department of Diagnostic and Interventional Radiology, Lausanne University Hospital, Lausanne, Switzerland; ^2^Département D'imagerie Médicale, CIUSSS du Nord-de-l'île-de-Montréal, Hôpital du Sacré-Cœur de Montréal, Montréal, QC, Canada; ^3^EEG Brain Mapping Core, Centre for Biomedical Imaging (CIBM) and Laboratory for Investigative Neurophysiology (The LINE), Department of Radiology, University Hospital Centre and University of Lausanne, Lausanne, Switzerland

**Keywords:** CT pulmonary angiography, pulmonary embolism, radiology technician, radiologist, interobserver agreement

## Abstract

**Objectives:** To assess interobserver variability between a trained radiology technician (RT) and an experienced radiologist in arterial obstruction quantification using the Qanadli obstruction index (QOI), in patients diagnosed with acute pulmonary embolism (APE) at CT pulmonary angiography (CTPA).

**Materials and Methods:** A RT and a radiologist independently reviewed CTPAs of 97 consecutive, prospectively enrolled patients with APE, and calculated the QOI. They classified patients into three risk categories: high for QOI ≥40%, intermediate for QOI 20–37.5%, low for QOI <20%. Interobserver variability was investigated for QOI as a continuous variable and as a categorical variable (high, intermediate, and low-risk groups).

**Results:** Mean QOI (±SD) was 39.5 ± 24.3% and 38.6 ± 18.9% for the RT and the radiologist, respectively. The mean QOI was not statistically different between the RT and the radiologist (*p* = 0.502), and the interobserver agreement was excellent (ICC = 0.905). The RT classified 54 patients (55.7%) as high, 17 (17.53%) as intermediate, and 26 (26.8%) as low risk. The radiologist classified 55 patients (56.7%) as high, 22 (22.7%) as intermediate, and 20 (20.6%) as low risk. The interrater agreement for risk stratification was excellent (weighted kappa = 0.844).

**Conclusion:** Once the diagnosis of APE was established, an adequately trained RT achieved an accuracy comparable to that of an experienced radiologist regarding QOI calculation and risk assessment.

## Introduction

Acute pulmonary embolism (APE) is a life-threatening condition associated with in-hospital mortality rates ranging from 5 to 75%, depending on the hemodynamic status (1–5). As a consequence, a specific diagnosis is needed for early risk stratification (RS) and appropriate management (3, 4, 6).

Echocardiography, although not recommended to diagnose suspected APE, is a useful tool to identify APE patients who have a poor prognosis (7, 8). However, echocardiography has several drawbacks, including occasional suboptimal image quality of the right ventricle (RV), and the inability to reliably demonstrate APE as the cause of RV pressure overload (1, 9, 10).

Today, contrast-enhanced multidetector computed tomography (CT), which enables the acquisition of high-resolution images covering the whole thoracic cavity within a single breath-hold, is the modality of choice for clinically suspected APE (1, 11–14). CT pulmonary angiography (CTPA) permits multiplanar reformatting, evaluation of pulmonary vessels till sub-segmental branches, and assessment of clot burden in the pulmonary arteries. Associated or alternative diagnoses can also be assessed with CTPA (9, 12, 15).

To evaluate the degree of vascular obstruction in APE, several quantitative scores have been described, including the modified angiographic Miller, and Walsh scores adapted for CTPA, and the CTPA-derived Mastora and Qanadli scores (16). To our knowledge, the Qanadli obstruction index (QOI) is the most used. The QOI is easy to calculate, even in cases with anatomical variations, and can provide an objective and reproducible means to quantify vascular obstruction, with a high degree of correlation to the selective pulmonary angiographic index (9, 11). Moreover, the QOI differentiates between complete and partial obstruction of the most proximal clot, which is supposed to add relevant details about lung perfusion (1).

Because APE is a critical medical condition, CTPA studies for suspected patients are frequently carried out in emergency departments which are commonly overcrowded, and proper management entirely relies on fast and accurate diagnosis (17). Although there may be concern about the role of trained radiology technicians (RT) in interpretative and quantitative radiological tasks, the unremitting workload increase and the shortage of radiologists have encouraged radiology departments to involve selectively trained RT in such tasks. Assigning this task to RT could, therefore, save radiologists' time to carry out more complex investigations (18, 19).

The primary purpose of our study was to compare the performance of a selectively trained RT with that of a radiologist in QOI score calculation and RS for patients with APE.

## Materials and Methods

### Patients

This prospective study included 97 consecutive patients aged between 65 and 93 years who presented clinically with signs and symptoms of APE and had positive CTPA findings. All CTPA scans were performed in a tertiary hospital between September 14th, 2009 and November 22nd, 2011. Uninterpretable CTPA due to suboptimal enhancement were excluded from the study (*n* = 5). All subjects were hemodynamically stable and were not in need of special care to maintain systolic blood pressure above 100 mmHg. All subjects gave written informed consent in accordance with the Declaration of Helsinki. The protocol was approved by the Ethics Committee of the Canton de Vaud.

### Image Acquisition

All CTPA scans were performed using a GE Healthcare Discovery CT750 HD (GE Healthcare, Milwaukee, WI, USA) following the radiology department's routine APE CTPA protocol. The acquisition was performed from the diaphragm to lung apices in supine position, during a single breath-hold or shallow breathing, depending on the patient's level of dyspnea. The images were obtained using 120 kVp, automatic exposure control enabled, 100–300 mA, beam collimation geometry of 64 × 0.625 mm, section thickness of 1.25 mm, reconstruction interval of 1 mm, and a table speed of 39.37 mm per 0.6 s rotation time (0.984 pitch). The field of view was appropriately adjusted to the size of the patient. A 70-mL bolus of iodine-based non-ionic contrast (Accupaque 300 mg/mL [Iohexol]; GE Healthcare, Oslo, Norway) was administered through an antecubital vein at a rate of 4 mL/s, followed by a 40 mL normal saline flush at a rate of 4 mL/s, and acquisition was triggered using the vendor's bolus tracking technique. Images were anonymized and de-identified before analysis and transferred to a workstation (Advantage Workstation 4.2, GE Healthcare, Buc, France). All axial images were reviewed using mediastinal window settings [window width [WW], 400 HU; window level [WL], 40 HU] and lung window settings (WW, 1,600 HU; WL, −600 HU). Observers were free to obtain sagittal and coronal maximum intensity projection (MIP) and to change window settings for optimal visualization of the vessels.

A RT who received a 3-month training on CTPA interpretation with QOI calculation under the supervision of a radiologist with 25 years of experience in cardiothoracic imaging, as well as a fellowship-trained vascular radiologist with 5 years of experience, interpreted the studies. The RT and radiologist were requested (a) to confirm the presence of partial or complete endovascular filling defects in the pulmonary arteries; (b) to calculate the QOI, and (c) to determine the RS category.

To calculate the QOI, each lung is considered to have 10 segmental arteries (three to the upper lobes, two to the middle lobe or lingula, and five to the lower lobes). When a proximal or lobar clot is present, occlusive disease does not need to be quantified in the vessels arising distally. The percentage of vascular obstruction is calculated as follows:

(1) Percentage ofobstruction(QOI)=[∑(n.d)/40]×100

where n is the number of segmental arterial branches arising distally (minimum, 1 indicates obstruction of one segment; maximum, 20 indicates obstruction of both the right and left pulmonary arteries) and d represents the degree of obstruction (minimum, 0 means patent vessel; maximum, two means occluded vessel). The value of d provides semi-quantitative information about the perfusion distal to the thrombus. Therefore, the maximum score is 40; to calculate the percentage of vascular obstruction (QOI), the patient score is divided by the maximal total score, and then the result is multiplied by 100. The RT and the fellowship-trained vascular radiologist were blinded to the patient records and independently interpreted the studies, quantified the QOI score, and then graded the risk (RS) based on the QOI score as follows: low risk, 1–17.5%; intermediate risk, 20–37.5%; high risk, ≥40% (20, 21).

### Statistical Analysis

Statistical analysis was conducted using R 3.1.3 (R Core Team, 2015, Vienna, Austria). Results are presented as absolute and relative number of subjects; quantitative variables are presented as mean ± standard deviation (SD). To investigate the difference between the QOI of the radiologist and the RT, a paired *t*-test was applied. The interobserver agreement was evaluated with intraclass correlation coefficients (ICC) and interpreted as follows: <0.40, poor; 0.40–0.59, fair; 0.60–0.74, good; ≥0.75, excellent. Differences in QOI quantification agreement were also visualized on a Bland-Altman plot. To investigate interobserver (RT vs. radiologist) agreement of qualitative ratings, weighted kappa coefficients were used and interpreted as follows: <0.01, poor; 0.01–0.20, slight; 0.21–0.40, fair; 0.41–0.60, moderate; 0.61–0.80, substantial; ≥0.81, excellent. *P*-values <0.05 were considered statistically significant.

## Results

The study population consisted of 97 patients with positive CTPA for APE, 57 (58.8%) males, and 40 (41.2%) females; mean age, 77.2 years; SD, 7.7 years.

**Figure 1:**
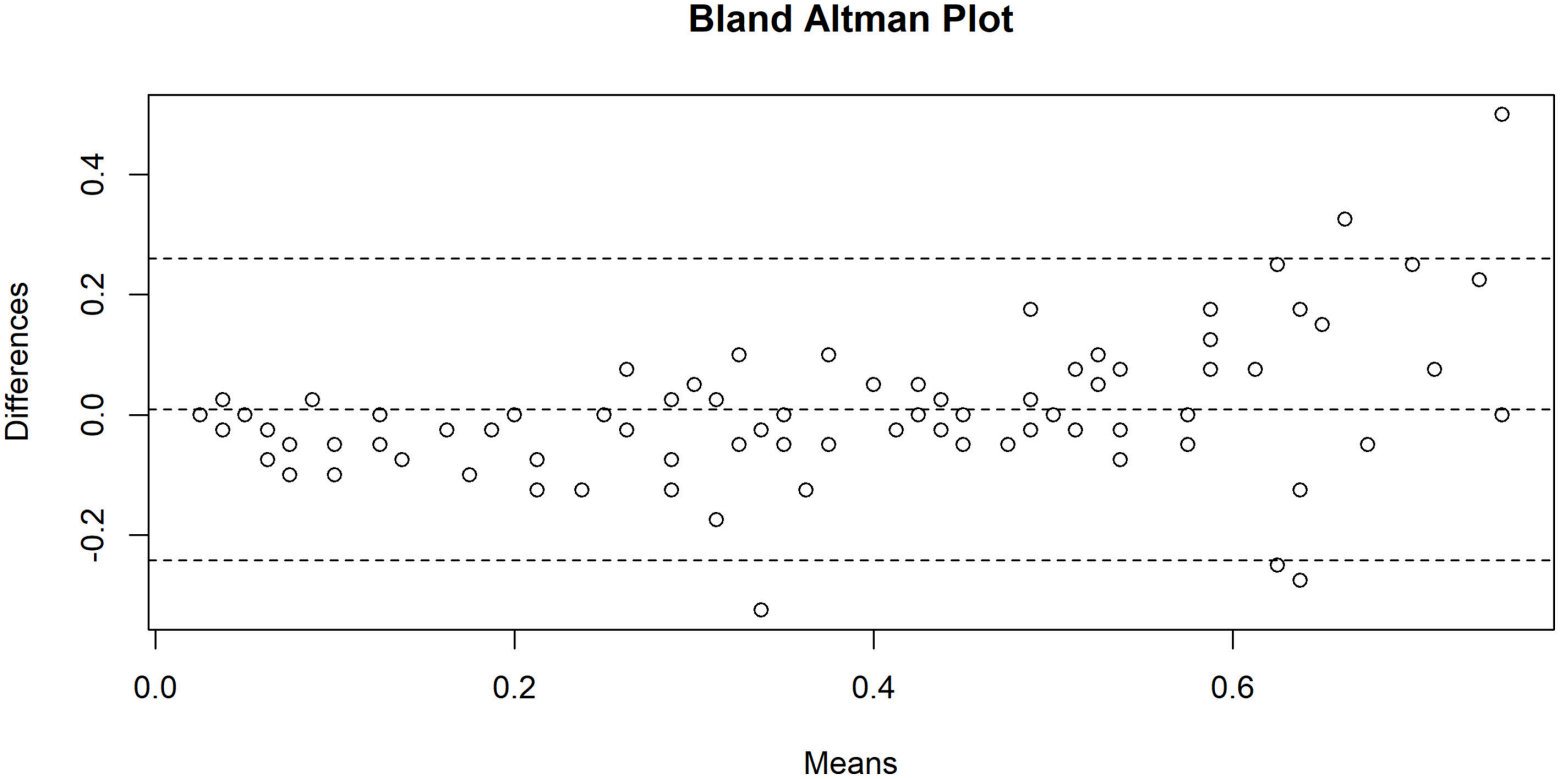
Bland-Altman plot showing agreement between Qanadli obstruction index (QOI) measurements by the radiology technician and the radiologist. The central dotted line indicates the mean of difference, the dotted upper and lower lines indicate the 95% limits of agreement. *N* = 97.

The mean overall QOI (±SD) was 39.5 ± 24.3% and 38.6 ± 18.9% for the RT and the radiologist, respectively. There was no statistically significant difference in mean QOI between the RT and the radiologist (*p* = 0.502), and the interobserver agreement was excellent (ICC = 0.905). Visual interpretation of the Bland-Altman plot confirms a good level of agreement between the RT and radiologist, with a trend towards a variability increase for higher QOI scores, i.e., in high-risk patients (Figure 1). The intra-observer agreement was excellent for the RT and the radiologist (ICC = 0.930 and 0.964, respectively).

Of the 97 CTPA studies included, the RT classified 54 (55.7%) patients as high risk, 17 (17.53%) as intermediate risk and 26 (26.8%) as low risk. On the other hand, the radiologist classified 55 (56.7%) patients as high risk, 22 (22.7%) as intermediate risk and 20 (20.6%) as low risk. The result of risk stratification by the RT and the radiologist is summarized in a three-way contingency table (see Table 1). The interobserver agreement was excellent (weighted kappa = 0.844). The intra-observer agreement was excellent for the RT and the radiologist (weighted kappa = 0.818 and 0.911, respectively).

**Table 1:** Three-way contingency table showing the risk-stratification agreement into low (QOI [Qanadli obstruction index] <20%), intermediate (QOI 20–37.5%) and high risk (QOI ≥40%) by the radiology technician vs. the radiologist.

		**Radiologist**			
		**Low**	**Intermediate**	**High**	**Total**
Radiology technician	Low	20	5	1	26
	Intermediate	0	14	3	17
	High	0	3	51	54
	Total	20	22	55	97

**Figure 2:**
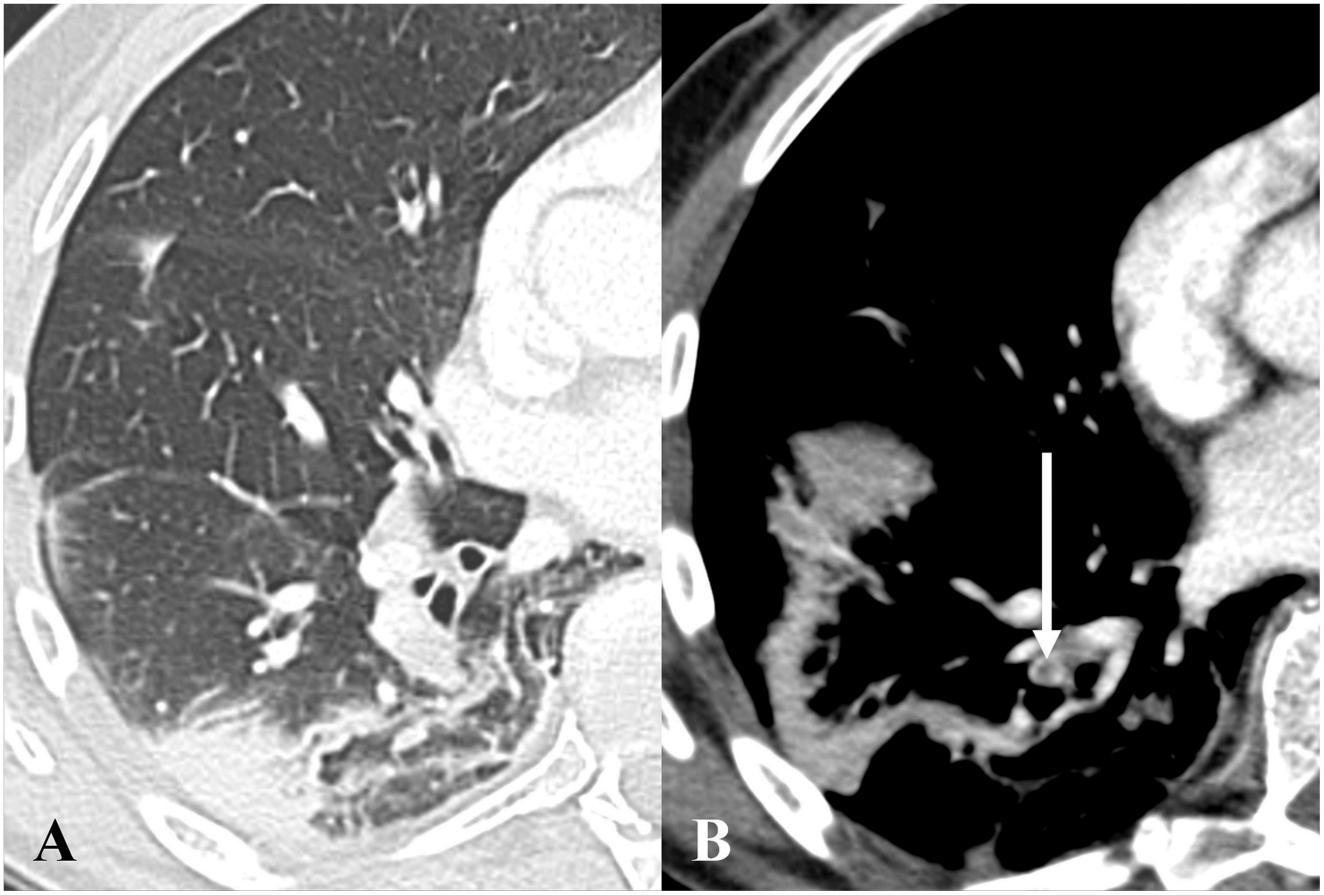
Axial CT pulmonary angiography in lung **(A)** and soft tissue windows **(B)**, in a 68-year-old male patient presenting with bilateral acute pulmonary embolism and associated basal atelectasis and pulmonary infarction. One of multiple emboli is depicted on **(B)** (white arrow). The radiology technician scored the Qanadli obstruction index (QOI) 35% with intermediate risk-stratification, while the radiologist scored the QOI 40% with high risk-stratification.

**Figure 3:**
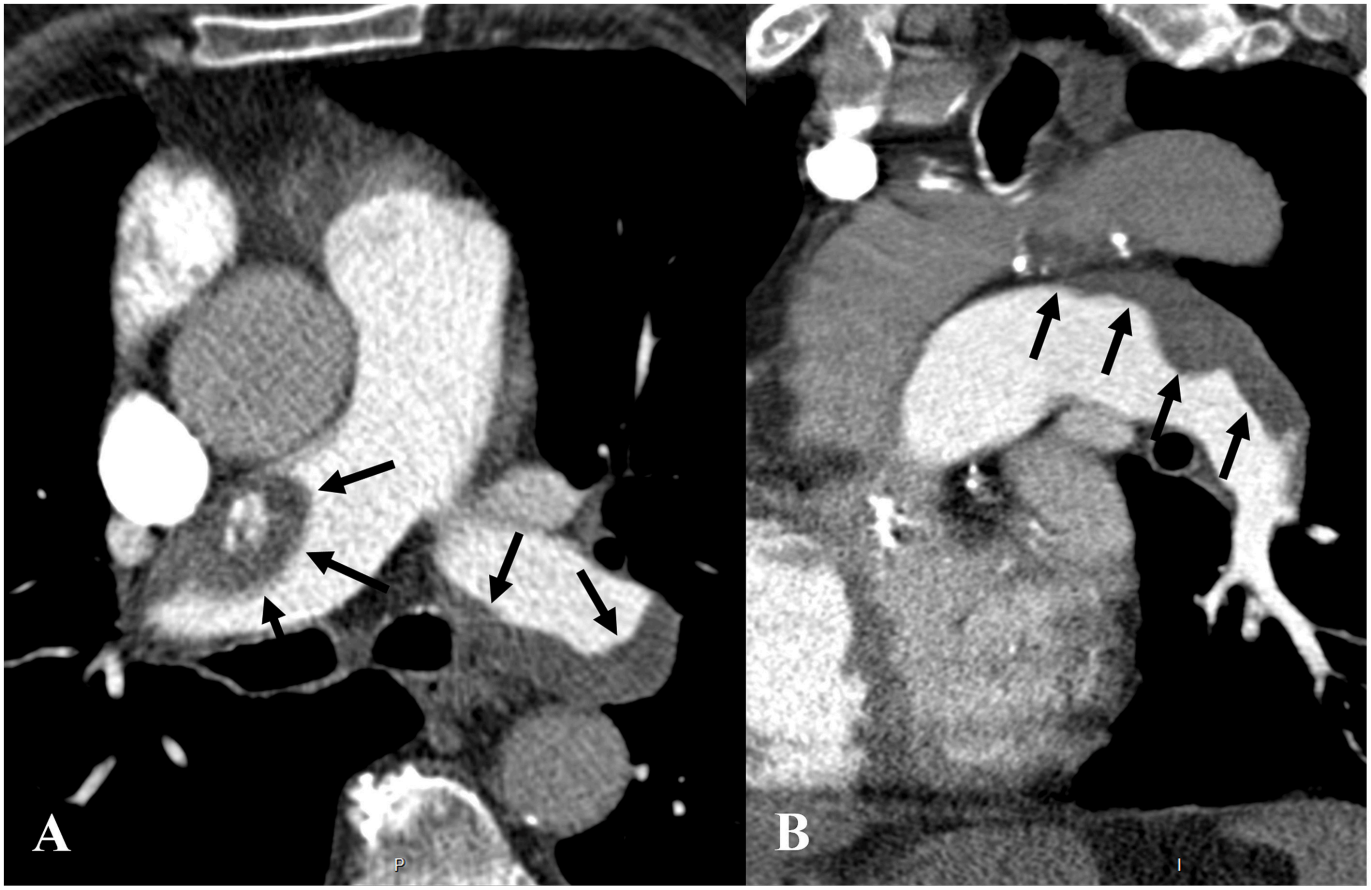
Axial **(A)** and sagittal oblique **(B)** CT pulmonary angiography in soft tissue windows in an 85-year-old man presenting with acute and chronic pulmonary embolism. **(A,B)** show flattened eccentric and mural thrombi (black arrows) with a broad base forming obtuse angles with the vessel wall in both pulmonary arteries, consistent with chronic pulmonary thromboembolism. The radiology technician rated the Qanadli obstruction index (QOI) 50% with high-risk stratification, while the radiologist rated the QOI 17.5% with low-risk stratification.

The discrepancy rate for RS was 11.3% (11 cases), of which 4 cases were related to artifacts (predominantly streak or metallic artifacts), 2 cases were related to motion artifacts, 2 cases were linked to adjacent pulmonary parenchymal consolidation due to infection and infarction/atelectasis (Figure 2), 1 case was referred to chronic pulmonary embolism (Figure 3). Only 2 cases (18.2%) had no apparent cause. We noticed that the QOI of the case with chronic pulmonary embolism was scored 50% by the RT and the RS assessment was categorized as high, while the radiologist scored the QOI 17.5% and RS assessment was categorized as low.

## Discussion

In this prospective study comparing quantitative CTPA interpretation between a trained RT and an experienced radiologist, we found no significant difference in QOI calculation and a high interobserver agreement in patient RS. Our results indicate that a selectively trained RT can detect APE on CTPA scans, quantify the QOI index and stratify patients' risk with satisfactory accuracy. In most cases of discrepancy, an underlying cause, such as artifacts or concomitant pulmonary pathology was found. It is noteworthy that one patient had findings consistent with chronic pulmonary embolism that let the RT overestimate the QOI, as chronic pulmonary emboli should not be included in the QOI.

In the last few decades, the diagnostic approach to suspected APE has dramatically changed due to substantial improvements in image quality of the pulmonary vasculature (14). CTPA currently is the modality of choice used to investigate patients with suspected APE because it is fast, readily available in most hospitals, cost-effective, allows for adequate visualization of pulmonary vessels and excludes alternative diagnoses. Estimated sensitivity and specificity for APE detection is 88 and 92–96%, respectively (1, 14, 22). Furthermore, several CTPA scores have been designed to objectively quantify pulmonary artery obstruction and provide a single-examination based RS. The QOI is one of these CTPA-derived scores that provides a simple, objective, and reproducible tool to quantify pulmonary vascular obstruction seen on CTPA. It may help select the appropriate therapeutic strategy not only for high-risk patients who require aggressive treatment and close surveillance but also to discharge low-risk patients who will benefit from outpatient management strategies. It has recently been found to be correlated to the clinical classification of pulmonary embolism severity as defined by the European Society of Cardiology (23). Finally, the QOI can be used to monitor treatment outcomes in follow-up scans (1, 2, 15, 24–29).

As APE is a life-threatening medical condition, CTPA for suspected APE is commonly performed in radiology departments during regular working hours or in the emergency department during night on-call hours. These departments are often facing many challenges because of the urgent nature of patients' conditions, overcrowding, the increasing workload as well as the shortage of radiologists (30). Subsequently, radiologists may take a longer time to read all radiological studies and precious time may be lost before final interpretation and diagnosis. Therefore, improving RTs' skills in simple quantitative reporting tasks could alleviate the reporting workload and free radiologists' time to perform other, more complex duties (31–34). Moreover, the involvement of RT in reporting would increase job satisfaction for the RT (30).

Recruiting RT to perform interpretative tasks in diagnostic radiology may be a matter of debate; nonetheless, several studies have shown that selectively trained RT can be involved in specific tasks of quantitative imaging. These studies have also suggested that carefully selected trained RT can achieve accurate performance comparable to that of radiologists (18, 19). To our knowledge, Swineburne was perhaps the first who proposed that RT can triage some types of radiologic films into normal or abnormal in 1971, highlighting the need for research in this field (35). Almost 15 years later, Berman et al. showed the potential of RT to improve radiologists' diagnostic performance (31). Jensch et al. compared the performance of RT and radiologists in the interpretation of CT colonography for colonic polyp detection and found good interobserver agreement, ranging from 71 to 91% (36). Debono et al. evaluated the accuracy of RT retrospectively re-reading screening mammograms, and they found sensitivity and specificity levels of 76.0–92.0 and 74.8–96.2%, respectively, suggesting that RT possess skills sufficient to read screening mammograms (37).

Brealey et al. retrospectively assessed the agreement between RT and radiologist accuracy in reporting of plain accident/emergency (A/E) and general practice radiographs, and its effect on clinician confidence in the diagnosis and management. They found that, for appendicular skeletal radiographs, the radiologist's accuracy was 87% and the RT's accuracy was 85%. The reporting accuracy on the remaining body radiographs for the radiologist and the RT were 85 and 84%, respectively. Also, this study showed that clinicians' confidence was more adversely affected when the radiologist's reports were incorrect than when RT's reports were incorrect. They also suggested that a carefully selected RT can accurately report both A/E and general practice plain radiographs (19).

Most of the previous research undertaken in this field is mainly focused on plain X-rays rather than computed tomography scans. As far as we know, there is no study specifically addressing the degree of agreement between a trained RT and a radiologist for CTPA interpretation. Our study is perhaps the first to compare the ability of a properly trained RT to calculate the QOI with an experienced cardiothoracic radiologist.

The main limitation of this study is that the reading of CTPA scans was limited to the QOI quantification process and patient RS. The ability of a trained RT to establish the correct diagnosis of APE was not evaluated. Furthermore, as designed, the study was not able to assess the effect of the learning curve on the interobserver agreement.

## Conclusion

Our study shows that once the diagnosis of acute PE is established, a dedicated RT trained in reading CTPA can fulfill tasks such as QOI calculation and risk stratification with a performance comparable to that of an experienced radiologist, with an excellent overall interobserver agreement. Discordance occurred because of chronic pulmonary clots, technical limitations and the presence of concomitant thoracic pathology. Nevertheless, this study provides evidence that trained RTs have the potential to quantify obstruction in CTPA with APE findings.

## Ethics Statement

This study was reviewed and approved by the ethics committee of the canton de Vaud. Written informed consent was obtained from every participant.

## Author Contributions

DR: study design, data analysis, and interpretation, literature review; SB: data acquisition, interpretation, literature review; J-FK: data analysis, statistical analysis; CB-A: data analysis, literature review; A-MJ: data acquisition and analysis; SQ: study design, data analysis, and interpretation, literature review; All authors contributed in drafting the manuscript and revising it critically.

### Conflict of Interest Statement

The authors declare that the research was conducted in the absence of any commercial or financial relationships that could be construed as a potential conflict of interest.

## References

[1] Attia NM Seifeldein GS Hasan AA Hasan A Evaluation of acute pulmonary embolism by sixty-four slice multidetector CT angiography: correlation between obstruction index, right ventricular dysfunction and clinical presentation. Egypt J Radiol Nucl Med. (2015) 46:25–32. 10.1016/j.ejrnm.2014.10.007

[2] Yu T Yuan M Zhang Q Shi H Wang D. Evaluation of computed tomography obstruction index in guiding therapeutic decisions and monitoring percutanous catheter fragmentation in massive pulmonary embolism. J Biomed Res. (2011) 25:431–7. 10.1016/S1674-8301(11)60057-2 23554721 PMC3596723

[3] Inönü H Acu B Pazarli AC Doruk S Erkorkmaz Ü Altunkaş A. The value of the computed tomographic obstruction index in the identification of massive pulmonary thromboembolism. Diagn Interv Radiol. (2012) 18:255–60. 10.4261/1305-3825.DIR.4597-11.4 22249888

[4] Varol K Gumus C Yucel H Sezer F Seker E Inci MF. Correlation of right ventricular dysfunction on acute pulmonary embolism with pulmonary artery computed tomography obstruction index ratio (PACTOIR) and comparison with echocardiography. Jpn J Radiol. (2015) 33:311–6. 10.1007/s11604-015-0419-0 25895158

[5] Konstantinov IE Saxena P Koniuszko MD Alvarez J Newman MA. Acute massive pulmonary embolism with cardiopulmonary resuscitation: management and results. Texas Hear Inst J. (2007) 34:41–5. 17420792 PMC1847913

[6] Becattini C Agnelli G. Acute pulmonary embolism: risk stratification in the emergency department. Intern Emerg Med. (2007) 2:119–29. 10.1007/s11739-007-0033-y 17619833

[7] Goldhaber SZ. Echocardiography in the management of pulmonary embolism. Ann Intern Med. (2002) 136:691–700. 10.7326/0003-4819-136-9-200205070-00012 11992305

[8] Pruszczyk P Goliszek S Lichodziejewska B Kostrubiec M Ciurzynski M Kurnicka K. Prognostic value of echocardiography in normotensive patients with acute pulmonary embolism. JACC Cardiovasc Imaging. (2014) 7:553–60. 10.1016/j.jcmg.2013.11.004 24412192

[9] Ghaye B Ghuysen A Bruyere PJ D'Orio V Dondelinger RF. Can CT pulmonary angiography allow assessment of severity and prognosis in patients presenting with pulmonary embolism? What the radiologist needs to know. Radiographics. (2006) 26:23–40. 10.1148/rg.261055062 16418240

[10] Reid JH Murchison JT. Acute right ventricular dilatation: a new helical CT sign of massive pulmonary embolism. Clin Radiol. (1998) 53:694–8. 10.1016/S0009-9260(98)80297-3 9766724

[11] Qanadli SD El Hajjam M Vieillard-Baron A Joseph T Mesurolle B Oliva VL. New CT index to quantify arterial obstruction in pulmonary embolism: comparison with angiographic index and echocardiography. Am J Roentgenol. (2001) 176:1415–20. 10.2214/ajr.176.6.1761415 11373204

[12] Méan M Tritschler T Limacher A Breault S Rodondi N1 Aujesky D. Association between computed tomography obstruction index and mortality in elderly patients with acute pulmonary embolism: a prospective validation study. PLoS ONE. (2017) 12:e0179224. 10.1371/journal.pone.0179224 28594950 PMC5464630

[13] Engelke C Rummeny EJ Marten K. Acute pulmonary embolism on MDCT of the chest: prediction of cor pulmonale and short-term patient survival from morphologic embolus burden. Am J Roentgenol. (2006) 186:1265–71. 10.2214/AJR.05.0650 16632717

[14] Metafratzi ZM Vassiliou MP Maglaras GC Katzioti FG Constantopoulos SH Katsaraki A. Acute pulmonary embolism: correlation of CT pulmonary artery obstruction index with blood gas values. Am J Roentgenol. (2006) 186:213–9. 10.2214/AJR.04.1320 16357404

[15] Vedovati MC Germini F Agnelli G Becattini C. Prognostic role of embolic burden assessed at computed tomography angiography in patients with acute pulmonary embolism: systematic review and meta-analysis. J Thromb Haemost. (2013) 11:2092–102. 10.1111/jth.12429 24134450

[16] Bankier AA Janata K Fleischmann D Kreuzer S Mallek R Frossard M. Severity assessment of acute pulmonary embolism with spiral CT: evaluation of two modified angiographic scores and comparison with clinical data. J Thorac Imaging. (1997) 12:150–8. 10.1097/00005382-199704000-00012 9179827

[17] Dolatabadi AA Baratloo A Rouhipour A Abdalvand A Hatamabadi H Forouzanfar M Interpretation of computed tomography of the head: emergency physicians versus radiologists. Trauma Mon. (2013) 18:86–9. 10.5812/traumamon.12023 24350159 PMC3860675

[18] Brealey S Scally A Hahn S Thomas N Godfrey C Coomarasamy A. Accuracy of radiographer plain radiograph reporting in clinical practice: a meta-analysis. Clin Radiol. (2005) 60:232–41. 10.1016/j.crad.2004.07.012 15664578

[19] Brealey SD King DG Hahn S Crowe M Williams P Rutter P. Radiographers and radiologists reporting plain radiograph requests from accident and emergency and general practice. Clin Radiol. (2005) 60:710–7. 10.1016/j.crad.2004.11.013 16038699

[20] Wu A Pezzullo J Cronan J Hou DD Mayo-Smith WW. CT pulmonary angiography: quantification of pulmonary embolus as a predictor of patient outcome–initial experience. Radiology. (2004) 230:831–5. 10.1148/radiol.2303030083 14739314

[21] Guo ZJ Liu HT Bai ZM Lin Q Zhao BH Xu Q. A new method of CT for the cardiac measurement: correlation of computed tomography measured cardiac parameters and pulmonary obstruction index to assess cardiac morphological changes in acute pulmonary embolism patients. J Thromb Thrombolysis. (2018) 45:410–6. 10.1007/s11239-018-1628-z 29417409

[22] Ginsberg MS King V Panicek DM. Comparison of interpretations of CT angiograms in the evaluation of suspected pulmonary embolism by on-call radiology fellows and subsequently by radiology faculty. Am J Roentgenol. (2004) 182:61–6. 10.2214/ajr.182.1.1820061 14684513

[23] Guo F Zhu G Shen J Ma Y. Health risk stratification based on computed tomography pulmonary artery obstruction index for acute pulmonary embolism. Sci Rep. (2018) 8:17897. 10.1038/s41598-018-36115-7 30559454 PMC6297138

[24] Venkatesh SK Wang SC. Central clot score at computed tomography as a predictor of 30-day mortality after acute pulmonary embolism. Ann Acad Med Singapore. (2010) 39:442–7. 20625619

[25] Konstantinides S V Torbicki A Agnelli G Danchin N Fitzmaurice D Galiè N 2014 ESC Guidelines on the diagnosis and management of acute pulmonary embolism. Eur Heart J. (2014) 35:3033–69. 10.1093/eurheartj/ehu283 25173341

[26] Konstantinides S V Torbicki A Agnelli G Danchin N Fitzmaurice D Galiè N Corrigendum to: 2014 ESC Guidelines on the diagnosis and management of acute pulmonary embolism. Eur Heart J. (2015) 36:2642 10.1093/eurheartj/ehu479 26224077

[27] Torbicki A Perrier A Konstantinides S Agnelli G Galiè N Pruszczyk P. Guidelines on the diagnosis and management of acute pulmonary embolism: the task force for the diagnosis and management of acute pulmonary embolism of the European Society of Cardiology (ESC). Eur Hear J. (2008) 29:2276–315. 10.1093/eurheartj/ehn310 18757870

[28] Furlan A Aghayev A Chang CC Patil A Jeon KN Park B. Short-term mortality in acute pulmonary embolism: clot burden and signs of right heart dysfunction at CT pulmonary angiography. Radiology. (2012) 265:283–93. 10.1148/radiol.12110802 22993221 PMC3447174

[29] Matsuoka S Kotoku A Yamashiro T Matsushita S Fujikawa A Yagihashi K. Quantitative CT evaluation of small pulmonary vessels in patients with acute pulmonary embolism. Acad Radiol. (2018) 25:653–8. 10.1016/j.acra.2017.11.013 29331359

[30] Renwick IG Butt WP Steele B. How well can radiographers triage x ray films in accident and emergency departments? BMJ. (1991) 302:568–9. 10.1136/bmj.302.6776.568 2021720 PMC1669393

[31] Berman L de Lacey G Twomey E Twomey B Welch T Eban R. Reducing errors in the accident department: a simple method using radiographers. Br Med J. (1985) 290:421–2. 10.1136/bmj.290.6466.421 3918612 PMC1417735

[32] Tamjeedi B Correa J Semionov A Mesurolle B. Interobserver agreement between on-call radiology resident and general radiologist interpretations of CT pulmonary angiograms and CT venograms. PLoS ONE. (2015) 10:e0126116. 10.1371/journal.pone.0126116 25938666 PMC4418836

[33] Safari S Baratloo A Negida AS Taheri MS Hashemi B Selkisari SH. Comparing the interpretation of traumatic chest x-ray by emergency medicine specialists and radiologists. Arch Trauma Res. (2014) 3:e22189. 10.5812/atr.22189 25738133 PMC4329230

[34] Branstetter BF Morgan MB Nesbit CE Phillips JA Lionetti DM Chang PJ. Preliminary reports in the emergency department: is a subspecialist radiologist more accurate than a radiology resident? Acad Radiol. (2007) 14:201–6. 10.1016/j.acra.2006.11.001 17236993

[35] Swineburne K Pattern recognition for radiographers. Lancet. (1971) 297:589–90. 10.1016/S0140-6736(71)91180-9 4100917

[36] Jensch S van Gelder RE Florie J Thomassen-de Graaf MA Lobé JV Bossuyt PM Performance of radiographers in the evaluation of CT colonographic images. AJR Am J Roentgenol. (2007) 188:W249–55. 10.2214/AJR.06.0451 17312031

[37] Debono JC Poulos AE Houssami N Turner RM Boyages J. Evaluation of radiographers' mammography screen-reading accuracy in Australia. J Med Radiat Sci. (2015) 62:15–22. 10.1002/jmrs.59 26229663 PMC4364802

